# Supplementary material for: Pharmacological Inhibition of IKK to Tackle Latency and Hyperinflammation in Chronic HIV-1 Infection
Source: Int J Mol Sci. 2022 Nov 30;23(23):15000. doi: 10.3390/ijms232315000 (PMC9741028; doi:10.3390/ijms232315000)
Supplement: Supplementary file 1 [file ijms-23-15000-s001.zip › ijms-2040113-Supplementary.pdf]

International Journal of
Molecular Sciences

MDPI

Supplementary materials

Table S1. Summary of IKK inhibitors included in the study.

Compound	Source	Cat. No.	Target	IC50	Ref.
MRT67307	Invivogen	inh-mrt	TBK-1/IKKε	19 nM (TBK1); 160 nM (IKKϵ)	[1]
BX-795	Invivogen	tlrl-bx7	TBK-1/IKKε	6 nM (TBK1); 41 nM (IKKε)	[2]
TBK1/IKKε-IN-1	Selleckchem	S8922	TBK-1/IKKε	1.0 nM (TBK1); 5.6 nM (IKKε)	[3]
TBK1/IKKε-IN-2	Selleckchem	S0425	TBK-1/IKKε	-	[4]
GSK8612	Selleckchem	S8872	TBK-1	158 nM	[5]
TPCA-1	Selleckchem	S2824	IKKβ	17,9 nM	
SC514	Selleckchem	S4907	IKKβ	3-12 μM	[6]

Table S2. Immunological and virological characteristics of study participants at the time of cell sample collection.

ID	Age	Sex	Ethnicity	Estimated min. length of HIV infection (years)	Estimated min. length of viral suppression (years)	CD4 Nadir (cells/μL)	CD4 count (cells/μL)	Viral Load (copies/mL)	ART Regimen*
1	38	M	Caucasian	4	4	460	932	<40	DRV/COBI
2	39	M	Caucasian	8	4.9	603	902	<40	DTG/ABC/3TC /DOVATO
3	36	F	Caucasian	7	6.4	504	1396	<40	ABC/3TC, DOVATO, RAL

* ABC, abacavir; COBI, cobicistat; DRV, darunavir; DTG, dolutegravir; RAL, raltegravir; 3TC, lamivudine.

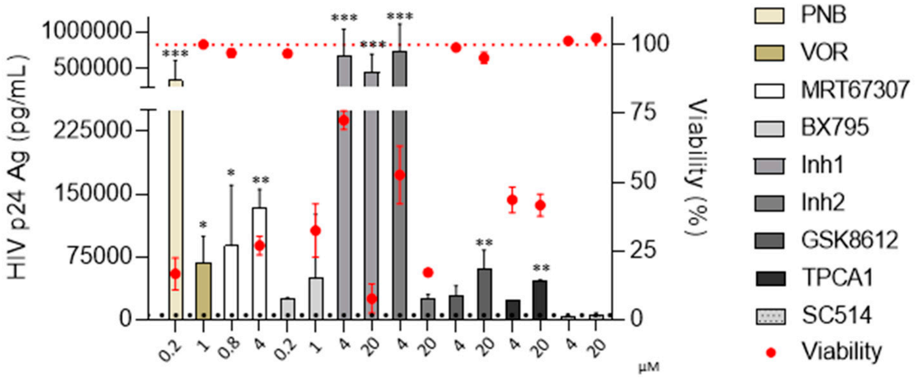

Figure S1. IKK Inhibitors induce HIV reactivation in *in vitro* lymphoid ACH-2 model of latency. HIV reactivation is determined by the expression of HIV-1 p24 antigen in cell culture supernatants as measured by ELISA in ACH-2 cultured for 48 h in the presence of subtoxic concentrations of IKKis. HDCAi panobinostat (PNB) and vorinostat (VOR) were used as controls for HIV-1 reactivation. Basal reactivation (grey-dashed line) in ACH-2 was established according to the non-drug condition (ND). Red dashed line represents toxicity in untreated condition. Mean ± SD of three independent experiments is shown. *p < 0.05; **p < 0.01, ***p < 0.001.

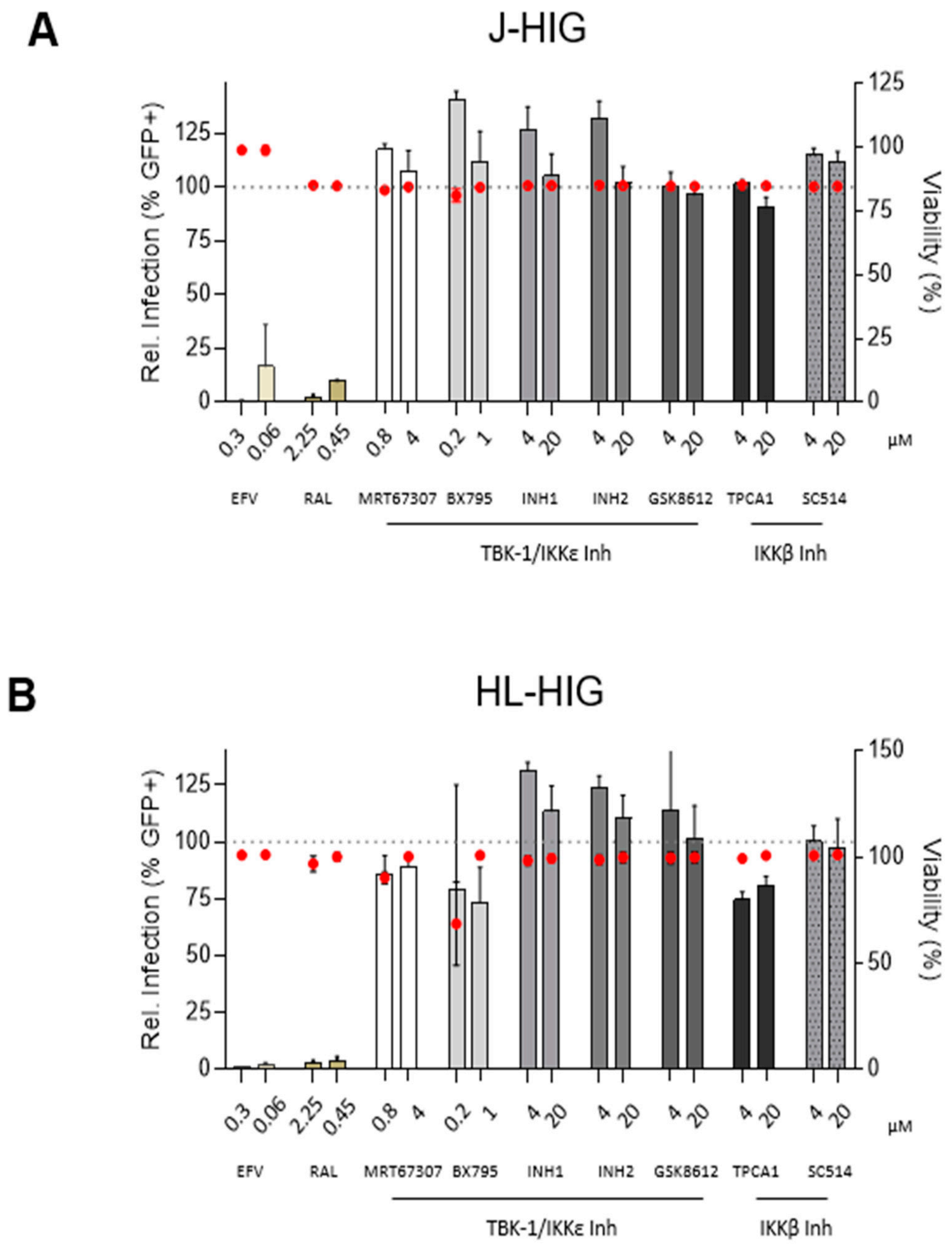

Figure S2. Antiviral activity of IKK inhibitors in acute HIV-1 infection cell models. IKKis do not exert anti-HIV activity on *in vitro* acute infection in lymphoid Jurkat (A) and myeloid HL-60 cells (B) as measured by the generation of GFP+ cells 72h after infection with VSV-HIG. Anti-HIV compounds efavirenz (EFV) and raltegravir (RAL) were used as controls. Grey-dashed line indicates infection level in ND control. Red dots indicate relative cell viability. Mean ± SD of three independent experiments is shown.

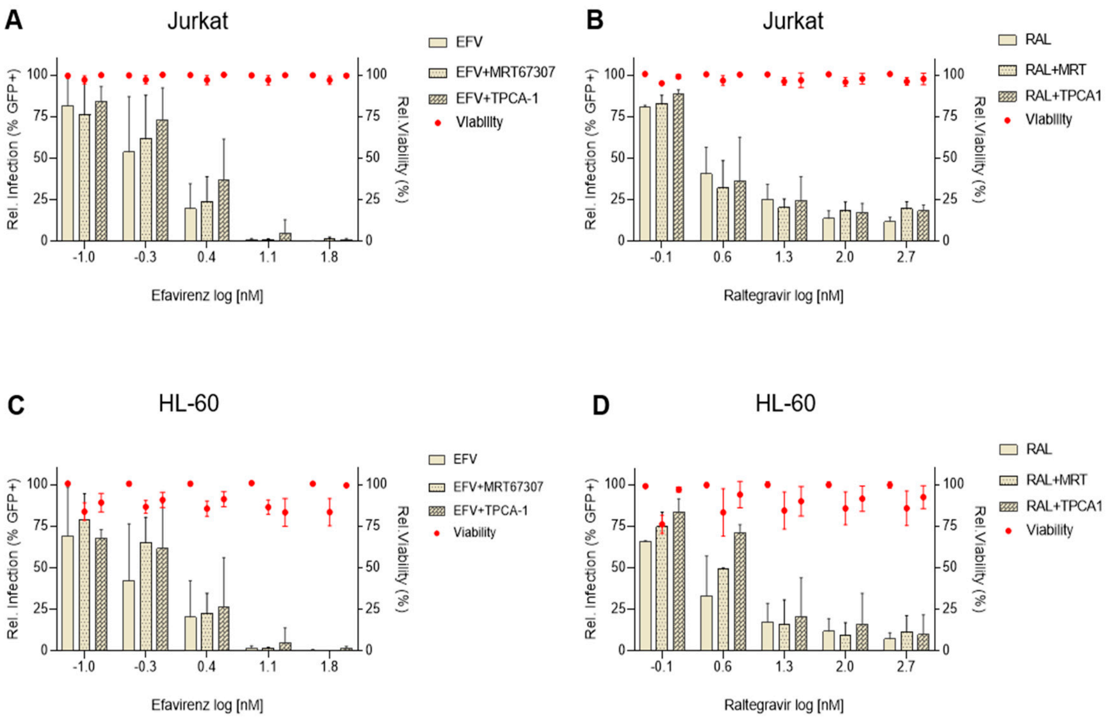

Figure S3. Antiviral activity of antiviral drugs in combination with IKK inhibitors. Anti-HIV activity of EFV (A) and RAL (B) in Jurkat cells coincubated with 4 µM of MRT67307 or TPCA-1 and infected with pseudotyped HIV-1 (VSV-HIG). Infection was measured as the percentage of GFP+ cells 48h after infection. Anti-HIV activity of EFV (C) and RAL (D) in HL-60 cells incubated with 4 µM of MRT67307 or TPCA-1. Red dots indicate cell toxicity of each IKKi alone. Mean ± SD of three independent experiments is shown.

References

1. Clark, K.; Peggie, M.; Plater, L.; Sorcek, R.J.; Young, E.R.R.; Madwed, J.B.; Hough, J.; McIver, E.G.; Cohen, P. Novel cross-talk within the IKK family controls innate immunity. *Biochem. J.* **2011**, *434*, 93–104. https://doi.org/10.1042/BJ20101701.
2. Clark, K.; Plater, L.; Peggie, M.; Cohen, P. Use of the Pharmacological Inhibitor BX795 to Study the Regulation and Physiological Roles of TBK1 and IκB Kinase Epsilon: A Distinct Upstream Kinase Mediates Ser-172 Phosphorylation and Activation. *J. Biol. Chem.* **2009**, *284*, 14136–14146. https://doi.org/10.1074/JBC.M109.000414.
3. Jenkins, R.W.; Aref, A.R.; Lizotte, P.H.; Ivanova, E.; Stinson, S.; Zhou, C.W.; Bowden, M.; Deng, J.; Liu, H.; Miao, D.; et al. Ex Vivo Profiling of PD-1 Blockade Using Organotypic Tumor Spheroids. *Cancer Discov.* **2018**, *8*, 196–215. https://doi.org/10.1158/2159-8290.CD-17-0833.
4. Muvaffak, A.; Pan, Q.; Yan, H.; Fernandez, R.; Lim, J.; Dolinski, B.; Nguyen, T.T.; Strack, P.; Wu, S.; Chung, R.; et al. Evaluating TBK1 as a Therapeutic Target in Cancers with Activated IRF3. *Mol. Cancer Res.* **2014**, *12*, 1055–1066. https://doi.org/10.1158/1541-7786.MCR-13-34-0642.
5. Thomson, D.W.; Poeckel, D.; Zinn, N.; Rau, C.; Strohmer, K.; Wagner, A.J.; Graves, A.P.; Perrin, J.; Bantscheff, M.; Duempelfeld, B.; et al. Discovery of GSK8612, a Highly Selective and Potent TBK1 Inhibitor. *ACS Med. Chem. Lett.* **2019**, *10*, 780–785. https://doi.org/10.1021/ACSMEDCHEMLETT.9B00027.
6. Kishore, N.; Sommers, C.; Mathialagan, S.; Guzova, J.; Yao, M.; Hauser, S.; Huynh, K.; Bonar, S.; Mielke, C.; Albee, L.; et al. A Selective IKK-2 Inhibitor Blocks NF-κ B-Dependent Gene Expression in Interleukin-1 Beta-Stimulated Synovial Fibroblasts. *J. Biol. Chem.* **2003**, *278*, 32861–32871. https://doi.org/10.1074/JBC.M211439200.
